# Authors’ Response to “Are Fourth Generation E-cigarettes Reducing Disease in the Population?”

**DOI:** 10.1093/ntr/ntae154

**Published:** 2024-06-24

**Authors:** Karin A Kasza, Zhiqun Tang, Young Sik Seo, Adam F Benson, MeLisa R Creamer, Kathryn C Edwards, Colm Everard, Joanne T Chang, Yu-Ching Cheng, Babita Das, Olusola Oniyide, Nicole A Tashakkori, Anna-Sophie Weidner, Haijun Xiao, Cassandra A Stanton, Heather L Kimmel, Wilson Compton, Andrew Hyland

**Affiliations:** Department of Health Behavior, Roswell Park Comprehensive Cancer Center, Buffalo, NY, USA; Behavioral Health and Health Policy, Westat, Rockville, MD, USA; Department of Health Behavior, Roswell Park Comprehensive Cancer Center, Buffalo, NY, USA; Center for Tobacco Products, US Food and Drug Administration, Silver Spring, MD, USA; National Institute on Drug Abuse, National Institutes of Health, Bethesda, MD, USA; Behavioral Health and Health Policy, Westat, Rockville, MD, USA; National Institute on Drug Abuse, National Institutes of Health, Bethesda, MD, USA; Center for Tobacco Products, US Food and Drug Administration, Silver Spring, MD, USA; Center for Tobacco Products, US Food and Drug Administration, Silver Spring, MD, USA; Center for Tobacco Products, US Food and Drug Administration, Silver Spring, MD, USA; Center for Tobacco Products, US Food and Drug Administration, Silver Spring, MD, USA; Center for Tobacco Products, US Food and Drug Administration, Silver Spring, MD, USA; Center for Tobacco Products, US Food and Drug Administration, Silver Spring, MD, USA; Center for Tobacco Products, US Food and Drug Administration, Silver Spring, MD, USA; Behavioral Health and Health Policy, Westat, Rockville, MD, USA; National Institute on Drug Abuse, National Institutes of Health, Bethesda, MD, USA; National Institute on Drug Abuse, National Institutes of Health, Bethesda, MD, USA; Department of Health Behavior, Roswell Park Comprehensive Cancer Center, Buffalo, NY, USA

We appreciate Dr. Glantz’s comment that our paper “Divergence in Cigarette Discontinuation Rates by Use of Electronic Nicotine Delivery Systems (ENDS): Longitudinal Findings From the United States PATH Study Waves 1–6”^1^ is well done.^2^ Our descriptive paper reports differences in real-world trends in population-level cigarette discontinuation rates across 2013/2014–2021, comparing US adults who smoked combustible cigarettes and used ENDS with US adults who smoked combustible cigarettes and did *not* use ENDS (regardless of other tobacco product use). We found that the relationship between adults’ ENDS use and cigarette discontinuation in the context of an expanded ENDS marketplace, new tobacco regulatory actions, and coronavirus disease 2019 (COVID 19) differs from the relationship in earlier years.

The richness of the PATH Study data, with its longitudinal design, large sample size, national representativeness, biomarker data, and detailed assessment of tobacco product use, is a great resource to address a wide range of questions relevant to tobacco research. As of December 2023, the research community has published 737 papers using the PATH Study data or methods.^3^

Dr. Glantz’s letter highlights research on a topic different from ours—assessing the disease impact of e-cigarettes through changes in cigarette smoking behavior. The unique longitudinal PATH Study design is advantageous for assessing longer-term patterns of e-cigarette exposure to predict new onset of disease while controlling for critical confounders like age, amount of past cigarette exposure, and recency of past cigarette exposure. This is important research to be done.

The PATH Study has made every effort to make all data sets and associated documentation available to all researchers as soon as possible. As of this writing, the Restricted Use Files are available to researchers through Wave 7 and the Public Use Files for Wave 7 will be available to all in late 2024. Linked exposure and potential harm biomarker data are also available to investigators. An array of documentation and training videos are available to help people make the best use of the PATH Study data resources. New data files and materials are continually being released. All of these materials are available to the research community through the National Addiction and HIV Data Archive Program (https://www.icpsr.umich.edu/web/NAHDAP/studies/36231). The PATH Study data can be used to address the question posed by Dr. Glantz in his letter and many other vital tobacco research questions—we encourage people to use the freely available data to do so.
